# Production of Recombinant Proteins in the Milk of Transgenic Animals: Current State and Prospects

**Published:** 2018

**Authors:** M. V. Shepelev, S. V. Kalinichenko, A. V. Deykin, I. V. Korobko

**Affiliations:** Institute of Gene Biology, Russian Academy of Sciences, Vavilova Str., 34/5, Moscow, 119334, Russia

**Keywords:** transgenic animal, recombinant protein, mammary gland, targeted genome editing, homologous recombination, site-specific recombination

## Abstract

The use of transgenic animals as bioreactors for the synthesis of the recombinant proteins secreted into milk is a current trend in the development of biotechnologies. Advances in genetic engineering, in particular the emergence of targeted genome editing technologies, have provided new opportunities and significantly improved efficiency in the generation of animals that produce recombinant proteins in milk, including economically important animals. Here, we present a retrospective review of technologies for generating transgenic animals, with emphasis on the creation of animals that produce recombinant proteins in milk. The current state and prospects for the development of this area of biotechnology are discussed in relation to the emergence of novel genome editing technologies. Experimental and practical techniques are briefly discussed.

## INTRODUCTION

For many years, genetically modified laboratory animals have been an effective tool for studying the functional properties of genes, proteins, and other molecules, and their importance as human disease models in biomedical research can hardly be overestimated. Such animals can be used to study the pathogenesis and molecular features of diseases, for the identification and validation of new therapeutic targets, and for effective search and development of new drugs, including preclinical studies. At the same time, genetically modified animals are becoming increasingly attractive objects in fields such as livestock farming, where genome changes can be used to correct economically important animal traits. Finally, transgenic animals can serve as bioreactors for the synthesis of the recombinant proteins secreted into milk, which enables the production of recombinant proteins in substantially larger amounts and at much lower costs than the production of proteins in eukaryotic cell cultures [1]. According to a prognosis by the RAND Corporation, an analytical company, which was published in 2006, the use of the mammary gland as a bioreactor for the production of recombinant proteins will be one of the most important areas of biotechnology to the year 2020 [2]. The prognosis is evidenced not only by numerous experimental studies in this direction, but also by already commercially available drugs based on recombinant human proteins. For example, recombinant human antithrombin III (Atryn®) is produced from the milk of transgenic goats, and a recombinant human C1 esterase inhibitor (Ruconest®) is obtained from rabbit milk [3]. In the last decade, revolutionary changes have occurred in the field of genome modification, due to the opportunity afforded by highly effective targeted genome editing and the significant simplification of this technology after the discovery of the CRISPR/Cas9 system. This has enabled the development of new approaches to the generation of animals, including economically important species whose milk contains recombinant proteins. New approaches will dramatically simplify and improve efficiency in the creation of such animals. In this review, we consider technologies for the generation of transgenic animals, with an emphasis on animals that produce recombinant proteins in milk. We outline today’s landscape and prospects in this field in terms of the emergence of new genome editing technologies and briefly describe experimental and practical studies.

## CLASSICAL TRANSGENESIS OF ANIMALS AND THE GENETIC ELEMENTS REQUIRED FOR THE PRODUCTION OF RECOMBINANT PROTEINS

The classical method for the generation of transgenic mammals, which was developed in the early 1980s and has been in wide use up to the present time, entails the microinjection of a transgene-containing DNA fragment into the pronucleus of a fertilized oocyte, followed by transplantation of the oocyte to competent (pseudo-pregnant) animals. In the scheme, the transgene-containing DNA fragment is randomly integrated into the recipient genome during natural processes of genomic DNA breakage and repair [4]. Transgene-containing linear DNA fragments, both intact and after nonspecific cleavage in the cell, can integrate into various sites of the genome. The number of transgene copies in the integration site also varies within a wide range [5]. In addition, the integration process can occur at various stages of embryo development, which leads to the mosaicism of primary transgenic animals: i.e., to the presence of a transgene not in all the cells of the organism. Obviously, generating a line of transgene-carrying animals requires the presence of a transgene in the genome of germ cells and inheritance of transgene-containing genomic DNA.

Therefore, during classical transgenesis, the transgene is randomly integrated into the recipient’s genome; in this case, the number of integrated transgene copies, including incomplete transgene fragments, is uncontrolled. If production of a recombinant protein is required, the transgenic construct should include a full expression module that provides autonomous transcription of the transgene in target tissues of the organism and proper mRNA processing, because of random transgene integration. When using alternative methods of transgenesis and technologies of targeted genome editing (see below), this requirement is not mandatory.

The key determinant that provides tissue specificity in transgene expression is the promoter. A number of promoters of genes encoding milk proteins have been successfully used for the production of recombinant proteins in the mammary gland. Promoters that enable production of the target protein at a sufficiently high level in milk (up to tens of grams per liter of milk) include promoters of goat and cow β-casein, cow α-s1-casein, rabbit whey acidic protein (WAP), human α-lactalbumin, and sheep β-lactoglobulin genes. However, the level of protein production depends not only on the promoter, but also on a number of other factors. In this case, promoters of one animal species can provide effective transcription of the transgene in the mammary gland cells of another animal species due to conservatism of the transcription factors regulating the production of milk proteins in mammary cells [6-19].

As the experience of generating transgenic animals has demonstrated, effective production of a recombinant protein often requires, apart from a tissue-specific promoter that ensures a high level of transgene transcription, inclusion of introns into the transgene. Inclusion of introns into the transgene in some experimental systems enabled a 400-fold increase in the transgene transcription level, compared to that from intron-free cDNA, while the effect of intron inclusion is minimal in other systems [20, 21]. Different introns placed in the same region of a gene may have opposite effects on the transgene expression level [21], and the same intron at different positions in the transgene may have opposite effects on the expression level [20, 21]. Introns, along with the possible inclusion of enhancers promoting high tissue-specific transgene transcription, as in the case of the first intron of the mouse β-casein gene [22], may also have an effect on the transgene expression level which is not related to transcription enhancement. One of the potential mechanisms of expression enhancement is regular arrangement of nucleosomes in the gene and the promoter region due to the presence of introns in the DNA sequence. A disruption of the nucleosome arrangement is supposed to disrupt initiation or elongation of transcription, complicating access to transcription factors or movement of RNA polymerase in the case of too closely located nucleosomes [23]. Another mechanism of intron-dependent enhancement of transgene expression may be the association between splicing and transcript polyadenylation [24]. Therefore, inclusion of introns in a transgene is generally considered as a way of increasing the level of transgene expression [25]. This fact determines the design of the protein-coding sequence of transgene that can be represented by cDNA, a full-length gene copy containing endogenous introns, or a mini-gene that includes either minimized native gene introns or hybrid/artificial introns [25-28]. In some cases, the use of a mini gene increases the transgene expression level in comparison with the cDNA as a transgene, providing simultaneous reduction in the overall size of the genetic construct compared to a full-length gene copy, thus simplifying handling of the transgene. It should be noted that, despite a significant amount of data on transgene design, there is no unambiguous and universal recipe for constructing a transgene coding sequence. Ideally, the creation of an animal that secretes a recombinant protein into milk should be accompanied by a comparative analysis of protein production using genetic constructs for transgenesis that contain cDNA, a full-length gene, and a mini-gene. However, such studies are undoubtedly associated with considerable costs.

Even the optimal expression cassette design does not guarantee effective transgene expression, which is due to the random site of transgene integration into the recipient genome. The surrounding chromatin, depending on the transgene integration site, can have a negative effect on transgene transcription. In addition, widespread tandem integration of several transgene copies can lead to suppression of their transcription due to transcriptional interference by neighboring copies [29]. Therefore, to increase the transgene expression level in classical transgenesis, the genetic construct often includes *cis*-elements that are designed to protect the transcription of the transgene from the influence of its environment. One of the most commonly used *cis*-elements is the chicken β-globin locus HS4 insulator [30, 31]. Inclusion of two tandem copies of the chicken β-globin locus HS4 insulator to the 5’-end of a genetic construct for transgenesis improves transgene expression but does not provide expression independent of the genomic integration site and the number of transgene copies [31, 32].

Therefore, the classical transgenesis used since the early 1980s has a number of significant drawbacks that are primarily due to the high variability of transgene expression caused by the randomness of its genomic integration site. Thus, to select an animal line with satisfactory parameters of recombinant protein production, a sufficiently large number of primary transgenic animals should be available. This may be a significant technical problem when generating transgenic livestock that produce recombinant proteins in milk, which is due to the need for a large number of embryos to generate a line of transgenic animals with satisfactory parameters of target recombinant protein production.

In addition to these drawbacks of classical transgenesis, the randomness of transgene integration into the recipient genome and uncontrolled variability in the number of transgene copies create certain difficulties specific to transgenic animals intended for practical use in the real economy. Namely, registration of modified organisms requires mandatory identification of the transformation event (the exact integration site of the transgenic construct into the genome) unique to the line of transgenic animals. In the case of classical transgenesis, identification of the transformation event for each transgenic animal line is a separate experimental problem whose solution is complicated if multiple transgene copies are integrated into the genome.

## ALTERNATIVES TO CLASSICAL TRANSGENESIS OF ANIMALS

The randomness of transgene integration into a recipient genome and uncontrolled variability in the number of transgene copies are significant drawbacks of the “classical” approach to the creation of transgenic animals. These drawbacks have stimulated the development of alternative technologies enabling transgene integration into a specific genomic site. Until recently, transgene integration into a specific genomic site using homologous recombination either in embryonic stem cells with a subsequent injection of genetically modified cells into blastocysts or in somatic cells, followed by somatic cell nuclear transfer (SCNT) into the oocyte, was the alternative to classical transgenesis. In both cases, genetic manipulations are performed with cells in culture, which enables a characterization of the accuracy of transgene integration before the generation of transgenic animals. In addition, a qualitative improvement of the classical transgenesis technology was a transgene integration into a pre-determined site of the genome, using homologous recombination through flanking of a transgenic expression cassette with genomic regions (“homology arms,” usually several thousands of nucleotide base pairs in length). It should be noted that the genetic elements enabling the production of a recombinant protein in milk are identical for the described approaches and classical transgenesis. The drawbacks of these approaches include the need for selective markers for picking cell clones with a genome-integrated transgene and a laborious clonal selection process that requires the analysis of a large number of cell clones (several hundreds or more) even when negative selection is used, which is due to the low efficiency of homologous recombination. In this case, even upon subsequent removal of a selective marker from the expression cassette, e.g., by means of site-specific recombination when the marker is flanked with appropriate recombination sites, exogenous DNA sequences, along with the target transgene sequences, inevitably remain in the genome, which may be undesirable.

Embryonic stem cells can be used as recipient cells for genetic manipulations *in vitro*. In this case, to generate a genetically modified animal, stem cells carrying a genetic modification are injected into blastocysts, with their subsequent implantation and creation of transgenic mosaic animals [33]. The descendants of animals containing the transgene in germinal cells will be nonmosaic transgenic animals. The disadvantage of this technology is a potential loss of pluripotent properties by stem cells upon generation of genetically modified clones during cultivation. In addition, the capabilities of this approach are substantially limited by the availability of embryonic stem cells of the target animal species. Generation of modified animals using stem cells has been used extensively in laboratory animals only. Somatic cells are an alternative to embryonic stem cells, allowing one to exclude the dependence on the preservation of pluripotent properties and enabling genetic modifications of virtually any animal species. In this case, the SCNT technology is used to produce transgenic animals by replacing the oocyte nucleus with somatic cell nucleus carrying the genetic modification and inducing embryo development. Despite the fact that epigenetic differences between the zygote genome and the somatic cell genome in this case do not significantly affect the traits of the produced organisms, the SCNT efficiency remains low. Animals are often unviable and die prematurely, which is associated with the side effects of somatic cell nuclear transfer, in particular with defects in the development of extraembryonic tissues and epigenetic reprogramming [34-36]. However, this particular technological approach was successfully used to generate goats secreting recombinant human antithrombin III (the basis for the approved Atryn® drug [37]) into milk, as well as several other lines of transgenic animals suitable for industrial use [8, 38, 39].

## SITE-SPECIFIC RECOMBINASES FOR TARGETED TRANSGENE INSERTION INTO THE GENOME

In addition to the methods based on homologous recombination of the transgene and on the use of cultured cells in combination with positive and negative selection for picking cell clones with homologous recombination, another alternative for targeted insertion of a transgene into a recipient genome is the use of site-specific recombinases. In general, the concept of usage of site-specific recombinases is based on the generation of a line of transgenic animals carrying recognition site(s) for recombinase in the genome. Such recognition sites can be integrated into a specific genomic site by means of homologous recombination or into a random genomic site by means of classical transgenesis. In the latter case, lines of transgenic animals with different variants of transgene localization in the genome are subjected to selection of a line with the transgene integrated into the genomic site that provides the required properties of transgene expression. This animal line is then used as a universal recipient for the insertion of different transgenes into a specific genomic site via site-specific recombination. For this purpose, a genetic construct containing a transgene flanked by recombination sites is microinjected into the fertilized egg of a transgenic animal containing the same recombination sites in the genome, together with a vector for the expression of recombinase or its mRNA [40, 41]. This results in site-specific recombination, and the transgene is inserted into the recipient genome. It is important to note that this can be done via microinjections directly into oocytes, in addition to the use of embryonic stem or somatic cell lines carrying recombinase recognition sites in their genome as recipients of the transgenic construct.

In practice, three recombination systems have been commonly used for site-specific transgenesis: phage P1 Cre recombinase, Flp recombinase of *Saccharomyces cerevisiae*, and phage φC31 integrase [42, 43]. In this case, the use of a native recombination site in a pair with its sequence-modified variant providing recombination only with a completely identical, but nonnative, recombination site enables insertion of the transgenic cassette in a predetermined direction – a recombinase-mediated cassette exchange (RMCE) technology [42, 44]. Integration of the cassette with recombination sites directly into the gene encoding the milk protein enables the expression of the target transgene, thus providing the production of the recombinant protein into the milk under the control of an endogenous promoter whose activity is specific to mammary cells [45]. The promoter of the β-casein encoding gene (gene for integration) [46-48] can be used for this purpose; lack of this gene does not affect normal lactation [49, 50].

An alternative approach to ensuring effective and stable transgene expression, in particular in mammary gland cells, using appropriate tissue-specific promoters is the use of so-called “safe harbors” as transgene integration sites. These harbors are genomic loci that are, on the one hand, insignificant for the development and functioning of the organism, which enables harmless transgene insertion into this locus and, on the other hand, provide a high level of transgene expression in the presence of appropriate regulatory elements in transgene. Examples of these genomic loci are the loci *ROSA26*, *Cd6*, *Hipp11*, and some others [33, 40, 51, 52].

In addition to the listed advantages, the use of site-specific recombinases and integrases for targeted transgene insertion into an animal genome has a regulatory significance in the case of transgenic animals intended for practical use, due to the significant simplification of the characterization of the transformation event (the site of transgene integration into the recipient genome).

## APPLICATION OF TARGETED GENOME EDITING TECHNOLOGIES IN ANIMAL TRANSGENESIS

The emergence of targeted genome editing technologies using site-specific nucleases has resulted in significant advances in the field of animal transgenesis that enable much more efficient transgene integration into a specific site of the recipient genome compared to the sole use of sequences for homologous recombination flanking the transgene [53]. Below, we discuss the molecular mechanisms underlying the targeted genome editing technologies that seems to be the most promising for generating economically important animals that secrete recombinant proteins into milk.

Targeted transgene integration using site-specific nucleases is based on a significant increase in the site-specific efficiency of transgene integration into the recipient genome during the repair of double- or single-strand DNA breaks [54]. Targeted genome editing technologies significantly increase the efficiency in transgene integration into a pre-determined site of the genome, which, in some cases, eliminates the use of selective markers and, most importantly, enables highly efficient targeted transgene integration directly into the genome of a zygote, followed by the generation of transgenic animals [55-57].

There are several classes of artificial nucleases that are used for targeted genome editing and the production of transgenic animals: zinc finger nuclease (ZFN) [58], transcription activator-like effector nuclease (TALEN) [59], artificial meganucleases [60], and hybrid artificial nucleases (e.g., mega-TAL [61], etc). However, the emergence of a targeted genome editing technology based on the CRISPR/Cas9 system was a revolutionary breakthrough in this field, due to the simplicity of its practical implementation in combination with high efficiency compared to TALEN and ZFN [53].

ZFN was the first nuclease designed for use in genetic engineering for targeted genome editing. The nuclease contains DNA-binding domains of the zinc finger protein (ZFP) that provides highly specific binding to the target DNA sequence, as well as the catalytic domain of FokI restriction endonuclease that introduces a double-strand break into the binding site. Each zinc finger recognizes a certain triplet of nucleotides. Three to six zinc fingers are used to construct the DNA-binding domain of ZFN. Their combination can be used to generate ZFN for almost any DNA sequence [62]. The structure and application of ZFN are described in detail in [63].

Later, a simpler, compared to ZFN, code of transcription activator-like effector (TALE) was deciphered [64, 65]. TALE proteins of pathogenic plant bacteria of the genus *Xanthomonas *contain a DNA-binding domain consisting of a series of monomers. Each monomer binds to one nucleotide in the target nucleotide sequence. Monomers are tandem repeats of 33–35 amino acid residues, except for the last “half-repeat” that consists of 20 amino acid residues. The amino acid residues located at positions 12 and 13 of the monomer are highly variable and responsible for recognizing a specific nucleotide: Asn-Ile, Asn-Gly, Asn-Asn, and His-Asp bind to the nucleotides A, T, G, and C, respectively. Like ZFN, artificial TALEN nuclease is a chimera of the TALE DNA-binding domain consisting of 20-30 monomers and the FokI nuclease catalytic domain [66], which introduces a double-strand break into the immediate vicinity of the target DNA sequence recognized by the variable amino acid residues of TALEN monomers.

In the CRISPR/Cas9 system, the target is recognized as a consequence of the complementary interaction between CRISPR RNA (crRNA) and the target DNA site. In this case, a complex of trans-activating crRNA (tracrRNA), crRNA, and Cas9 nuclease is assembled and then a double-strand break is introduced into a RNA/DNA duplex by Cas9 nuclease [67]. Therefore, the specificity and targeted action of a nuclease in the CRISPR/Cas9 system require only the synthesis of RNA that is complementary to the target genomic DNA. In contrast, ZFN and TALEN-based technologies often require a complex and labor-intensive protein design. To date, a number of modifications and analogues of the CRISPR/Cas9 system have been developed, e.g., CRISPR/Cpf1 and CRISPR/C2c2 [68-72] with improved properties for editing the genome and solving certain target goals.

It should be noted that the artificial nucleases ZFN and TALEN do not possess absolute specificity. To a larger extent, this problem relates to the CRISPR/Cas9 system. The problems of DNA cleavage by artificial nucleases in non-targeted genomic sites can be solved in various ways that allow for increasing the specificity of the changes introduced into the genome and reducing the probability of unprogrammed genetic changes. However, the problem of nonspecific modifications in the recipient genome is not so critical upon generation of transgenic animals as compared to targeted genome editing technologies in the field of clinical applications, since accidental changes that occurr in non-targeted genome sites can be excluded during breeding.

In eukaryotic cells, double-strand breaks introduced by site-specific nucleases can be repaired through several mechanisms; in particular by homologous-directed repair (HDR), where a repair template is the sister or homologous chromatid as well as the donor DNA with 200–800 bp homology arms [73], which enables DNA integration between the homology arms into the site of the genomic DNA break [74]. In addition, DNA repair can be achieved by means of non-homologous end-joining (NHEJ), where non-homologous or low-homologous (2–5 nucleotides) ends are ligated, which may lead to deletions or insertions of several nucleotides in length [75]. Repair can also occur by means of microhomology-mediated end-joining (MMEJ), which requires 5- to 25-bp homologous DNA at or near the break and leads to deletions, insertions, and translocations [76], as well as by means of single-strand annealing (SSA), which requires 30-bp or more homologous single-strand templates [77].

The key mechanism for the repair of breaks introduced by site-directed nucleases into a specific site of the genome is homologous recombination that enables integration of the transgene located between homology arms into a specific genomic site. This approach has been successfully implemented in animal transgenesis using double-strand templates containing a transgene flanked by 1- to 3-kb homology arms, with a repair efficiency of 0.5 to 20% [77-80]. Because the NHEJ mechanism is highly efficient in the repair of breaks introduced by artificial nucleases (up to 80%) [81], one of the ways to increase efficiency in homologous recombination and transgene insertion is by using NHEJ inhibitors [82, 83] that, however, have a mutagenic effect and increase the likelihood of transgene insertion into a non-targeted genomic locus [80]. Efficiency in homologous recombination can be increased by elongation of homology arms and by selection of optimal concentrations of components of the CRISPR/Cas9 system for genome modification microinjected into a zygote [78], as well as by using mutant Cas9 nuclease (nCas9, nickase) introducing distant single-strand DNA breaks into the plus and minus chains of a target genomic locus [84].

At the same time, alternative technologies have been developed which ensure targeted integration of extended (up to 10–15 kb) DNA fragments into a pre-determined genomic site without using the HDR mechanism. For example, one of such technologies exploits NHEJ-based repair of breaks by ligating the complementary overlapping single-strand DNA ends of the genomic target site and the repair template comprising a transgene, which are generated by a ZFN nuclease pair upon cleavage of the targeted sequences of the genome and repair template [85, 86]. Insertion of extended DNA fragments into a pre-determined genomic site of the double-strand breaks introduced by TALEN or Cas9 nucleases can also occur via the MMEJ mechanism, when the homologous recombination template includes short sequences homologous to the DNA fragments adjacent to the nuclease cleavage site [87, 88].

Therefore, the rapid development of targeted genome editing technologies has allowed researchers to avoid a number of the drawbacks inherent to classical transgenesis: e.g., random transgene integration into the genome and uncontrolled variability in the number of transgene copies. The CRISPR/Cas9 technology enables the generation of transgenic animals with transgene integration into a specified genomic site, which, together with the use of homologous recombination, determines the controlled number of transgene copies. In particular, one of the most promising approaches to the generation of animals producing recombinant proteins in milk is the CRISPR/Cas9-targeted integration of the transgene into the genes that encode milk proteins in such a way that transgene expression is controlled by the endogenous regulatory sequences of the recipient gene. Application of these technologies will simplify and standardize the technologies for generating transgenic animals for the production of recombinant proteins. This will make the transgenesis process more efficient and reduce costs in the generation of economically important transgenic animals. Genome editing technologies will allow researchers to generate transgenic animals with one transgene copy integrated into a specific genomic site, which will enable a reliable comparison of the influence of certain genetic elements present in a construct for transgenesis on recombinant protein production in milk, which cannot be done using classical transgenesis due to the integration of an uncontrolled number of transgene copies into different genomic loci in different lines of transgenic animals.

## CONCLUSIONS

The development of targeted genome editing technologies has opened new prospects for the generation of transgenic animals at a whole new level. The standardization of the generation of transgenic animals with specified and stable target traits is becoming possible thanks to the use of knowledge on the molecular genetic mechanisms of regulation of gene expression and genome functioning, as well as the available technologies of genetic engineering. That is fully applicable to the production of recombinant proteins in milk for the manufacturing of pharmaceuticals, biologically active additives, etc.

Taking into account the set of technologies generated to date, which are still under active development, the optimal direction of studies in the field of recombinant protein production in the milk of economically important animals points to the creation of animal lines (depending on the need in a target protein – rabbits, sheep, goats, and cows) whose genome is modified by the insertion of sequences for asymmetric directed recombination of the expression cassette into a milk protein-encoding gene (e.g., the β-casein gene). The promoter, and other regulatory sequences, of this gene will provide a high level of transgene expression. This insertion can be achieved with high efficiency through a microinjection into the oocytes of a genetic construct carrying an expression cassette, together with the corresponding recombinant integrase or its mRNA. These animal lines can be created with previously inaccessible efficiency by means of genome editing technologies us ing the CRISPR/Cas9 system or its analogues, thanks to the simplicity of implementation and design of this system. At the same time, the technologies of synthetic biology allow one to use mini-genes with artificial introns as a transgene, but not full-length gene copies, to facilitate efficient expression of the transgene and production of the target protein, thus simplifying the design and creation of genetic constructs for transgenesis.

Prospects for the development of such a direction in the production of recombinant proteins, primarily for medical needs, are supported by the two marketed drugs that are based on recombinant proteins obtained from the milk of transgenic animals. It should be noted that there is the possibility of producing significant quantities of recombinant proteins at costs substantially lower than those required for production in cellular systems. The use of modern technologies significantly simplifies compliance with regulatory requirements for describing the transformation event. At the same time, the requirements to the biological safety of recombinant protein production in milk will require a revision of the standards for the welfare of farm animals and veterinary control to exclude the presence of zoonotic and anthropozoonotic infectious agents, as well as controlled parameters of the manufactured drugs.

## Abbreviations

CRISPR: clustered regularly interspaced short palindromic repeat
HDR: homology-directed repair
MMEJ: microhomology-mediated end joining
NHEJ: non-homologous end joining
RMCE: recombinase-mediated cassette exchange
SCNT: somatic cell nuclear transfer
SSA: single strand annealing
TALE: transcription activator-like effector
TALEN: transcription activator-like effector nuclease
ZFN: zinc finger nuclease
